# Pioneering role of RNA in the early evolution of life

**DOI:** 10.1590/1678-4685-GMB-2024-0028

**Published:** 2024-09-02

**Authors:** Israel Muñoz-Velasco, Adrián Cruz-González, Ricardo Hernández-Morales, José Alberto Campillo-Balderas, Wolfgang Cottom-Salas, Rodrigo Jácome, Alberto Vázquez-Salazar

**Affiliations:** 1 Universidad Nacional Autónoma de México, Facultad de Ciencias, Departamento de Biología Celular, Mexico City, Mexico.; 2 Universidad Nacional Autónoma de México, Facultad de Ciencias, Departamento de Biología Evolutiva, Mexico City, Mexico.; 3 University of California Los Angeles, Department of Chemical and Biomolecular Engineering, California, USA.

**Keywords:** RNA world, ribozyme, origin of life, RNA viruses, viroids

## Abstract

The catalytic, regulatory and structural properties of RNA, combined with their extraordinary ubiquity in cellular processes, are consistent with the proposal that this molecule played a much more conspicuous role in heredity and metabolism during the early stages of biological evolution. This review explores the pivotal role of RNA in the earliest life forms and its relevance in modern biological systems. It examines current models that study the early evolution of life, providing insights into the primordial RNA world and its legacy in contemporary biology.

## Introduction

Mentioning heredity today almost automatically brings DNA to mind. Historically, the determination that DNA was the molecule responsible for heredity can be traced to Griffith’s experiments in 1928 (Griffith, 1928), the Avery-MacLeod-McCarty experiment in 1944 (Avery *et al.*, 1944), and the Hershey-Chase experiment in 1952 (Hershey and Chase, 1952). A year later, the discovery of the double helix structure by Watson and Crick established the framework for the central dogma of molecular biology, defining the flow of genetic information from DNA to RNA to protein, placing DNA at the highest and most prestigious position in biochemistry (Watson and Crick, 1953a, b; Crick, 1958; 1970; Cobb, 2017). However, this narrative underwent a significant paradigm shift with a series of findings that included the discovery of messenger RNA (mRNA) in the 1960s (Brenner et al., 1961; Gros et al., 1961), as well as its proposal as a genetic regulator (Jacob and Monod, 1961), the discovery of catalytic RNA molecules (ribozymes) in the 1980s (Kruger et al., 1982; Guerrier-Takada et al., 1983), the development of RNA *in vitro* evolution techniques in the 1990s (Ellington and Szostak, 1990; Tuerk and Gold, 1990), and the description of a long list of regulatory non-coding RNAs (ncRNA) over the last decades (Fu, 2014; Delihas, 2015). These milestones have elevated RNA to a much more important level, recognizing its crucial roles in cellular processes, genetic regulation, and evolutionary biology.

## Chemical and structural properties of DNA and RNA

Both DNA and RNA are nucleotide polymers, where each nucleotide consists of three fundamental components: a nitrogenous base (also known as a nucleobase), a pentose sugar, and a phosphate group. The nucleobases are derived from heterocyclic compounds, specifically purines (adenine and guanine) and pyrimidines (cytosine and thymine in DNA, or uracil in RNA). Nucleic acids have two types of pentose sugars (in beta-furanose form), which are covalently joined to a nucleobase by an N-β-glycosyl bond (at N-1 in pyrimidines and N-9 in purines). RNA contains D-ribose, whereas DNA features 2’-deoxy-D-ribose (Yoffe et al., 2008). Nucleotides are covalently linked together through phosphate-group bridges via a phosphodiester linkage, in which the 5’-phosphate group of a nucleotide is joined to the 3’-hydroxyl group of the next. This structure results in a phosphate backbone, whose ionization confers a negative charge to both DNA and RNA.

**Figure 1 -:**
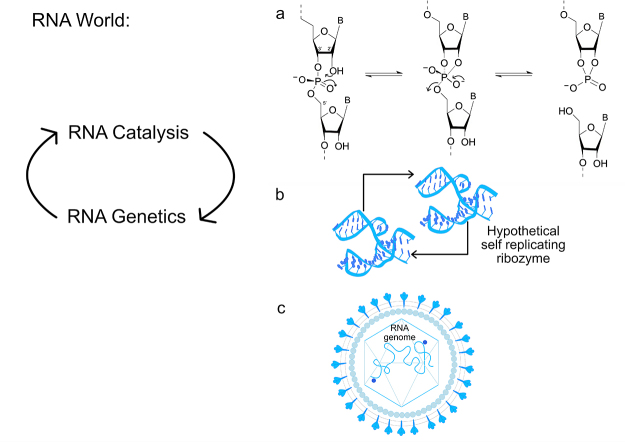
Roles played by RNA in catalysis and genetics. The RNA world hypothesis posits that early life on Earth primarily used RNA for catalysis and genetic information storage, predating DNA and proteins. a) The 2’-hydroxyl group in RNA facilitates phosphoryl transfer reactions like transesterification, in which the 2′-oxygen’s nucleophilic attack on an adjacent phosphate forms an unstable pentacoordinated phosphorus, forming a 2′,3′-cyclic phosphate product and a 5′-oxyanion. b) A self-replicating ribozyme was vital for establishing an RNA-based genetic system, marking a key evolutionary transition to an RNA world capable of Darwinian evolution. c) RNA functions as the genetic material in some viruses and viroids.

There are two main differences that distinguish DNA and RNA. The first one lies in their chemical components, while the second involves the three-dimensional structure they adopt in space. In the case of the chemical components, DNA contains thymine, whereas RNA contains uracil (Leslie and Grover, 2022). Although occasionally, DNA may have uracil (Sire et al., 2008), and RNA may have thymine in the form of ribothymidine (Clark et al., 2019). Compared to thymine, uracil has no methyl group in the C5’ carbon, making it less stable and susceptible to mutations (Vértessy and Tóth, 2009; Roberts and LaBonte, 2023). However, the main difference in components is the pentose sugar, which defines the identity of each nucleic acid. As mentioned above, RNA contains a D-ribose, with a hydroxyl (OH-) group in the C2’ position. This chemical group is tremendously reactive compared to the hydrogen in 2’-deoxy-D-ribose. In fact, the 2’ hydroxyl group endows RNA with the ability to catalyze phosphoryl transfer reactions, particularly transesterification or hydrolysis of phosphate esters (Figure 1 A ). These reactions involve the nucleophilic attack of the 2′-oxygen towards the neighboring phosphate atom, this phosphoester transfer generates a pentacoordinated phosphorus species that is not stable, resulting in the formation of a 2′,3′-cyclic phosphate compound and the liberation of a second product featuring a 5′-hydroxyl group (Emilsson et al., 2003). While transesterification is important for RNA catalytic activities involving self-cleavage and splicing (Lilley, 2003; 2011), it can also affect the stability of RNA, especially at high pH, where spontaneous degradation occurs (Adams et al., 1992; Emilsson *et al.*, 2003). 

Regarding their three-dimensional structure, DNA consists of two regular and stable helical chains wound around the same axis, forming a double helix that is held together by hydrogen bonds. Hydrogen bonding occurs in the same RNA chain, generating sequence-dependent secondary structure elements that are important in RNA function and stability. Some of these elements include stems, helices, bulges, and hairpin loops (Nowakowski and Tinoco, 1997; Anderson-Lee et al., 2016). Using these motifs, as well as its interactions with ions, RNA can adopt stable and complex three-dimensional structures, such as helical duplexes (Matsumoto and Sugimoto, 2021), major and minor groove triplexes (Devi et al., 2015), A-minor motifs (Baulin, 2021), triple-stranded structures (Brown, 2020), quadruplexes (Xu and Komiyama, 2023; Zareie et al., 2024), among many others (Butcher and Pyle, 2011). All the functions that RNA performs are closely linked to its ability to form these three-dimensional structures, including intricate quaternary complexes like the ribosome (an RNA heterotrimer) (Butcher and Pyle, 2011; Jones and Ferré-D’Amaré, 2015; Ganser et al., 2019).

Another important aspect to consider when discussing RNA structure and functionality is that several chemical modifications are often mistakenly identified as RNA adducts-and therefore as chemical damage-rather than as necessary modifications for the correct folding, regulation, and function of RNA. For example, 1, *N* ^*6*^ -dimethyladenosine (m^1^,^6^A)*,* is a modified nucleobase present in some mammalian transfer RNAs (tRNAs) that regulates processes related to cancer development (You et al., 2022). Another interesting RNA modification is *N* ^*1*^ -methyladenosine (m^1^A), which is present in mRNAs and regulates their structure and translation efficiency under stress conditions (Qi et al., 2023). These are just a few examples of the fascinating diversity of RNA beyond the canonical nucleobases, which inevitably lead to questions about the potential role of modified RNA molecules in early evolution.

## Exploring the evolutionary importance of RNA in biological systems

Although some researchers began to recognize the importance and antiquity of RNA molecules during the first half of the 20th century, after the emergence of molecular biology, most of the research efforts in biochemistry focused on understanding gene and enzyme functions, placing DNA and proteins as the most important molecules in the cell. It seemed that RNA was simply a mere intermediary between these two macromolecules. Nevertheless, as mentioned previously, the paradigm changed when the diverse roles of RNA molecules in crucial cellular processes such as DNA replication, gene expression, and regulation started to be discovered (Darnell, 2011).

## RNA as an early genetic polymer

The discovery that the infectivity of the tobacco mosaic virus resides in its RNA opened doors to speculation about its role in the early evolution of life. Notable researchers such as John B. S. Haldane, Jean L. A. Brachet, Andrey N. Belozerzky, and John D. Bernal concluded that RNA likely preceded DNA as the genetic molecule during the early stages of cellular evolution (Lazcano, 2016). Nevertheless, as discussed in later sections, the ability of RNA to store genetic information in some viruses does not necessarily imply that viruses are ancestral entities. Instead, it enriches its biological importance and emphasizes its versatility and adaptability.

The same year that Watson and Crick elucidated the double helix structure of DNA, Stanley Miller reported the first synthesis of organic molecules under conditions resembling the atmosphere of the early Earth (Miller, 1953). This synthesis was accomplished by applying an electric discharge to a combination of hydrogen, methane, ammonia, and water. The conditions were based on Harold Urey’s calculations (1952) and simulated a reducing atmosphere, an idea originally proposed by Oparin (Oparin, 1938). In his experiment, Miller reported amino acids such as glycine, α-alanine, β-alanine, α-amino-n-butyric acid, plus hydroxy acids, and urea (Miller, 1953). However, none of the nucleic acid components were obtained in his experiment. It was not until the early 1960s that Joan Oró synthesized adenine from the polymerization of hydrogen cyanide (Oró, 1960; Oró and Kimball, 1961). This finding was crucial in demonstrating that RNA components could be synthesized under possible early Earth conditions. Since then, significant efforts in prebiotic chemistry have focused on synthesizing the building blocks of RNA. Notable achievements include the synthesis of activated pyrimidine nucleotides from glycolaldehyde, cyanamide, and phosphate (Powner et al., 2009), and the synthesis of purine nucleotides from formamidopyrimidines (Becker et al., 2016). The simultaneous prebiotic synthesis of pyrimidine and purine nucleosides (mono- and diphosphates) from cyanoacetylene under wet-dry cycles has been reported recently (Becker *et al*., 2019). Remarkably, hydrogen cyanide and formamide, produced through electric discharges and laser-driven plasma impacts in a reducing atmosphere of NH_3_, CO, and H_2_O, can generate RNA nucleobases when exposed to UV light at high temperatures (Ferus et al., 2017). Similarly, experiments using a reducing atmosphere and borosilicate yielded a complete set of biological nucleobases, including RNA nucleobases (Criado-Reyes et al., 2021). Furthermore, the discovery of both pyrimidine and purine nucleobases in carbonaceous chondrites (Botta and Bada, 2002; Rios and Tor, 2013; Oba et al., 2022; Krishnamurthy et al., 2022) implies that these molecules were available in the solar system and were likely delivered to early Earth during meteorite collisions.

This body of research is essential for understanding the prebiotic era, marked by the synthesis and accumulation of organic compounds, including RNA nucleobases. During this period, chemical evolution facilitated the transformation of simple molecules into complex organic compounds through prebiotic reactions (Oró, 1983; Jia et al., 2021). This process provided the necessary precursors for the emergence of RNA as an early genetic polymer, and for crucial molecules, such as the proposed RNA-dependent RNA polymerase ribozyme, capable of catalyzing its own replication and the replication of other ribozymes (Figure 1 B ) (Joyce, 2002; Joyce and Szostak, 2018; Papastavrou et al., 2024). 

Although there are still many unsolved issues related to the synthesis and stability of RNA components and RNA molecules (as discussed in later sections), the evidence discussed in this review offers supports to the proposal of a pre-DNA era where RNA played a central role in the earliest cells, a concept known as the RNA world hypothesis.

## The RNA world(s)

Many proposals on the origin of life are based on the hypothesis that, following a period of prebiotic synthesis and accumulation of organic compounds, RNA molecules played a conspicuous role in both the replication of genetic material and catalysis, an epoch commonly known as the RNA world (Gilbert, 1986; Joyce, 2002). In broad terms, the concept of the RNA world refers to a set of models that seek to explain a hypothetical period in the early evolution of life on Earth, where ribonucleic acid molecules played a central role. The presence of biological entities endowed with RNA as genetic material, the astonishing *in vitro* expansion of the catalytic repertoire of RNA molecules, and the manifold roles that RNA molecules and ribonucleotides play in extant cells, provide a strong support to the RNA world hypothesis (Chen and Li, 2007; Lazcano, 2012, 2014; Vázquez-Salazar and Lazcano, 2018; Hernández-Morales et al., 2019). However, as discussed by Robertson and Joyce (2012), the RNA implies different premises for different researchers, with each holding their own interpretation of what it represents, thus creating not one but several RNA worlds. It is worth noting that Thomas Cech has already introduced and discussed the concept of ‘RNA worlds’ (Cech, 2012). Cech uses this plural form to refer essentially to three distinct RNA worlds: the primordial RNA world, the contemporary RNA world, and the world of RNA technology and medical applications. The latter two refer to the contemporary era which, given the recent discoveries in catalytic RNA, mRNA, and regulatory non-coding RNA, is an RNA-dominated world.

In this review, the RNA world is considered a stage in the early evolution of life where genetic information continuity was ensured by RNA self-replication. These primitive biological systems (ribocells) depended on RNA and its interactions with metal ions, minerals, and a broad catalog of organic molecules to carry out the chemical reactions necessary for maintaining their metabolism. This catalog may have included compounds capable of forming membranes, ribonucleotides, amino acids synthesized on primitive Earth or delivered by meteorites, and even small peptides, all of which could have contributed to shaping the chemical environment in which life evolved (Jadhav and Yarus, 2002; Hsiao et al., 2013; Wieczorek et al., 2013; Vázquez-Salazar and Lazcano, 2018; Müller et al., 2022).

## Historical perspective

The independent works of Carl Woese (1967), Francis Crick (1968), and Leslie Orgel (1968) in the late 1960s are often acknowledged as the seminal basis for formulating the RNA world hypothesis. The conclusions reached by these three authors are similar, suggesting that the earliest organisms on Earth used RNA as a genetic polymer and, possibly, as a catalyst before ribosomal protein synthesis. Yet, even before these bold proposals, biochemists such as Alexander Rich (1962), Philip Handler (1963), and Robert Eakin (1963) recognized the importance of ribonucleic acid and its ribonucleotide derivatives in primitive stages of metabolism, proposing that modern biochemistry is the result of evolution from an ancestral state where RNA and modified ribonucleotides were the protagonists.

### The discovery of ribozymes and the consolidation of an evolutionary hypothesis

In the early 1980s, Thomas R. Cech, from the University of Colorado Boulder, was investigating the *in vitro* splicing mechanism of one of the precursors of ribosomal RNA (rRNA) in the protist *Tetrahymena* (Cech *et al*., 1981). The initial hypothesis of his group was that a protein was catalyzing the editing of rRNA. However, they discovered that the folded structure of the RNA in the intron, as well as the involvement of a guanosine nucleotide and Mg^2+^ were sufficient to carry out the excision activity (Kruger et al., 1982). Cech and his colleagues categorized these self-splicing RNA molecules as group I introns, and coined the term ribozyme to define RNAs that are catalytically active.

Meanwhile, at Yale University, the group led by Sydney Altman was investigating the maturation mechanism of transfer RNA in bacterial organisms (Guerrier-Takada et al., 1983). In this maturation process, the tRNA molecule undergoes cleavage at its 5’ end site, which was known to be catalyzed by the RNAse P, a ribonucleoprotein (RNP) complex whose protein portion was postulated to be catalytic. However, Altman and his colleagues demonstrated that the nucleic acid portion of the RNAse P was responsible for catalysis.

These groundbreaking discoveries provided crucial evidence for understanding how RNA could have performed essential biochemical functions independently of proteins in early stages of life.

### The resurgence of the RNA world

In the foreword to the first edition of the book *The RNA World* (Gesteland and Atkins, 1993), Francis Crick wrote: “*This hypothesis of an RNA world without protein was largely forgotten but has now become fashionable again because of the remarkable discoveries by Altman and by Cech*.” Why was the proposal forgotten? Orgel and Crick (1993) answered this question by arguing that neither of them thought that RNA would continue to play a decisive role in the modern cell. Nevertheless, the finding of catalytic RNA molecules provided, almost inadvertently, the experimental evidence needed to bolster the theoretical proposals of Woese, Rich, Crick, and Orgel, and all the (r)evolutionary biochemists before them.

In addition to the ribozymes described by Cech and Altman, several naturally occurring ribozymes have been discovered. In 1986, a new type of self-splicing introns, termed group II introns, was independently reported by two groups (Peebles et al., 1986; van der Veen et al., 1986). These introns are less prevalent than group I and are commonly found in organellar and bacterial genomes. In the same year, other catalytic RNAs were discovered in plant pathogens with small circular RNA (circRNA) genomes. These ribozymes, named hammerhead (Hutchins et al., 1986; Prody et al., 1986) and hairpin (Buzayan et al., 1986), catalyze self-cleavage via transesterification reactions (de la Peña et al., 2017).

Other examples of naturally occurring ribozymes include the hepatitis delta virus (HDV) ribozyme, found in 1988, which catalyzes site-specific self-cleavage with a double-nested pseudoknot fold (Kuo et al., 1988). HDV-like ribozymes, identified in 2006, are structurally related to HDV ribozymes and include sequences from mammalian CPEB3, retrotransposons, and bacteria (Salehi-Ashtiani et al., 2006). Other notable small ribozymes include the Varkud satellite (VS) ribozyme, the largest known self-cleaving ribozyme, discovered in 1990 (Saville and Collins, 1990), and the *glmS* riboswitch, a self-cleaving ribozyme activated by glucosamine-6-phosphate, so far the only example of a natural ribozyme that uses a small organic cofactor for catalysis (Winkler et al., 2004; Ferré-D’Amaré, 2010). Recent discoveries include the twister ribozyme (Roth et al., 2014), the twister-sister, hatchet, and pistol ribozymes (Weinberg et al., 2015), and the hovlinc ribozyme found in human very long intergenic noncoding (vlinc) RNAs (Chen et al., 2021), all of which are nucleolytic ribozymes.

Besides naturally occurring ribozymes (products of biological evolution), various laboratories worldwide have successfully selected an extensive catalog of artificial ribozymes. These laboratory ribozymes (results of *in vitro* evolution experiments) display a variety of catalytic activities not found in the repertoire of biological RNAs (Wilson and Szostak, 1999; Martin et al., 2015). 

One recent example of *in vitro* selected ribozymes is the lineage of RNA-dependent RNA polymerase ribozymes, which descended from the class I ligase (Bartel and Szostak, 1993; Ekland et al., 1995) and are capable of amplifying RNA (Horning and Joyce, 2016). Continuous work on the *in vitro* evolution of the polymerase ribozyme made it capable of copying and amplifying multiple different RNA templates, albeit with modest fidelity (Tjhung et al., 2020). Building on this, Papastavrou et al. (2024) further evolved the ribozyme, demonstrating that it not only replicates RNA sequences for other ribozymes with high fidelity but also evolves over time, producing new variants with increased evolutionary fitness. This process mimics natural selection at the molecular level, providing evidence that early RNA molecules could undergo Darwinian evolution.

Collectively, both natural and artificial ribozymes can be classified under the six classes of catalytic activities present in protein enzymes: hydrolases, oxidoreductases, lyases, transferases, ligases, and isomerases (Hernández-Morales et al., 2019; Müller et al., 2016; Wilson and Lilley, 2021; Deng et al., 2023; Fine and Pearlman, 2023). A diverse catalog of ribozymes enhances the appeal of the RNA world hypothesis, allowing the envision of a scenario where a ribocell, devoid of protein enzymes, could sustain a protometabolism.

## Relics of the RNA world in the modern cell

RNA plays a wide variety of roles in the modern cell. For instance, it is involved in genome replication, transcription, protein translation, regulation of gene expression, and metabolism control (Cech and Steitz, 2014). It has been proposed that some of these functions could have originated in the RNA world. Among these, perhaps the most surprising is protein synthesis, performed in all living cells by the ribosome (cf. Noller, 2012). This ribonucleoprotein complex represents one of the most ancient molecular machineries of the cell (Petrov et al., 2014).

It was precisely the interest in protein synthesis that led to the early recognition of the significant role played by RNA in this process, as experimental measurements showed a correlation between the amount of RNA in cells and their protein synthesis capacity (Caspersson, 1941; Brachet; 1942). However, it was not until the biochemical description of the microsomal fraction of the cytosol (Claude, 1946) that an association between RNA and proteins in the translation process was established (Keller et al., 1954). In 1954, George Palade used electron microscopy to morphologically characterize granules composed of RNA and proteins (Palade, 1955), which were later named ribosomes (Hartman, 1959). Since then, the evolution of the translation apparatus has captured the attention of countless scientists (Woese, 1965; Crick, 1968; Orgel, 1968).

Biochemical approaches aimed to understand the peptide bond formation pointed towards a possible involvement of ribosomal RNA in this process (Noller and Chaires, 1972). However, it was the pioneering work of Ada Yonath (Yonath et al., 1980) that standardized the preparation and availability of quality ribosome crystals, enabling the molecular-level scrutiny of the RNA-protein interactions that constitute this ribonucleoprotein complex (Ban et al., 1999; 2000; Cate et al., 1999; Clemons et al., 1999; Nissen et al., 2000; Schluenzen et al., 2000; Wimberly et al., 2000). The most significant conclusion eventually reached is the recognition that the catalytic center of the ribosome, where the peptide bond forms and which is highly conserved across all domains of life, is entirely constituted by RNA (cf. Moore and Steitz, 2011); that is, the ribosome is a ribozyme (Cech, 2000). Evolutionarily, this evidence suggests that protein synthesis originated in an RNA world as a result of a very early process (Fox, 2010).

With the study of the role of RNA in the evolution of life, it was also proposed that certain molecules present in modern metabolism are, in fact, molecular fossils of catalysts dating back to the RNA world. Although this idea had already been contemplated in the 1960s by Handler (1963), Eakin (1963), and Orgel (1968), it was not until 1976 that Harold White developed a comprehensive hypothesis on the evolution of contemporary metabolism from an ancestral one (White, 1976).

Similar to Handler and Eakin, White was interested in the chemical structure of coenzymes, which play a crucial role in the catalytic functionality of a large number of enzymes (White, 1976; 1982). White observed that several coenzymes possess a ribonucleotide motif in their chemical structures, which could be attributed to an early diversification process in the catalytic capabilities of RNA. Considering the presence of coenzymes in living organisms, the dependency of enzymes on these molecules, and the fact that many coenzymes are ribonucleotides or derivatives thereof, White proposed that these molecules are the molecular fossils of an ancestral metabolic state (White, 1976; 1982).

White proposed a scheme where modern metabolism is the result of an evolutionary process where protein enzymes were preceded by enzymes made from nucleic acids (White, 1976; 1982). Particularly, the ribonucleotides present in the catalytic sites of ribozymes were the portions conserved through evolution, found in contemporary metabolism as coenzymes of a ribonucleotide nature. This evolutionary reasoning was supported by experimental results demonstrating that some coenzymes, particularly thiamine (Mizuhara and Handler, 1954), could catalyze reactions by themselves similar to those catalyzed in the enzymatic systems where they participate (White, 1976).

The RNA world might have also driven the evolution of molecules crucial for chemical signaling and environmental sensing. These molecules, known as alarmones, include ribonucleotides and their derivatives. Alarmones remain essential in modern cells and are synthesized under stress conditions (Stephens et al., 1975), triggering a stringent response (Stent and Brenner, 1961) that affects major cellular processes such as genome replication, gene expression, and metabolism (Nelson and Breaker, 2017; Hernández-Morales et al., 2019). The widespread distribution of the enzymes that biosynthesize alarmones suggests that these molecules were present in ancestral populations before the divergence of the Archaea, Bacteria, and Eukarya domains (Lazcano et al., 2011; Hernández-Morales *et al*., 2019). The ribonucleotide structure of alarmones, their extensive biological distribution, functional role in highly conserved cellular processes, presence in meteorites and prebiotic experiments, and the possibility of synthesis by ribozymes all support the proposal that these modified nucleotides are molecular fossils from the RNA world. During this period of evolution, RNA molecules, ribonucleotides, and their derivatives were essential not only for catalysis and genetic information transfer but also for chemical signaling and metabolite sensing (Hernández-Morales *et al*., 2019).

## RNA diversity in modern biological systems

**Figure 2 -:**
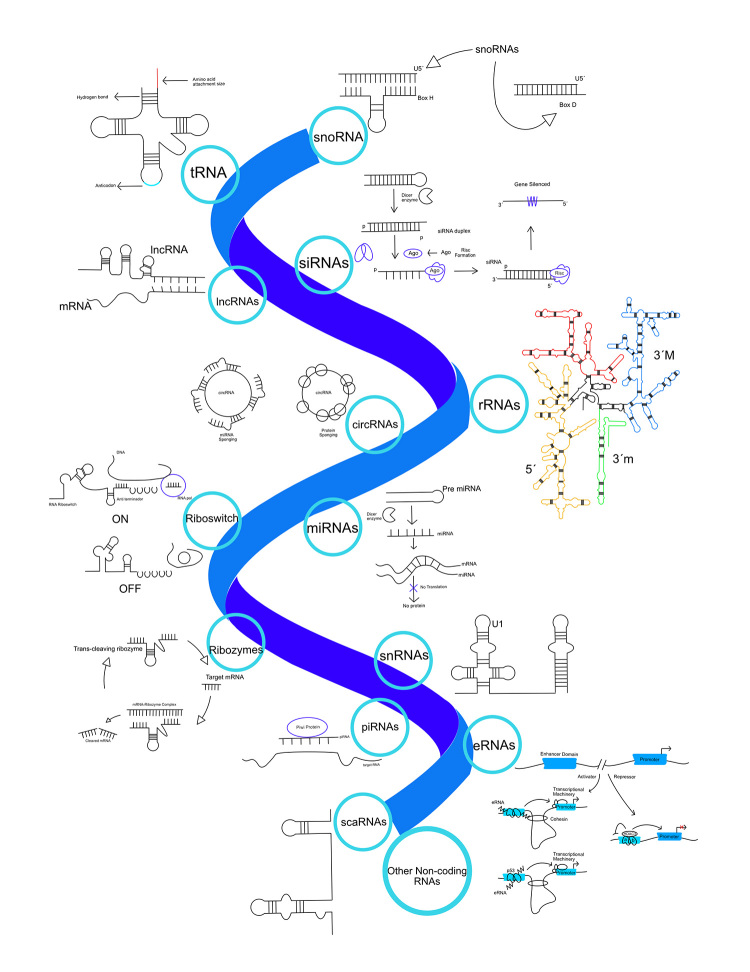
Overview of RNA molecules found in modern biological systems. The diversity of RNA molecules is illustrated; many of these molecules play crucial biological roles in the Eukarya cellular domain (e.g. regulating gene expression).

In addition to its well-known role in protein synthesis (rRNA, tRNA, mRNA), RNA also serves various non-coding functions. Eukaryotes contain a plethora of non-coding RNAs, some of which are also present in prokaryotes (Figure 2). Furthermore, some of these RNAs are believed to have originated and played significant roles during the RNA world, as discussed below.

Long non-coding RNAs (lncRNAs) are RNA molecules normally exceeding 200 bases in length. Similar to other ncRNAs, lncRNAs lack protein-coding capacity, and their structure is characterized by modularity and an abundance of sequence repeats (Mattick et al., 2023). They are broadly classified into five categories based on the locations of their transcripts: 1) intergenic, 2) intronic, 3) sense, 4) antisense, and 5) bidirectional (Ma *et al*., 2013). lncRNAs play crucial roles in cellular functions, such as recruiting RNA-protein complexes to target genes. They act as decoys by binding and sequestering regulatory proteins away from their target DNA sequences (Mattick *et al*., 2023). Additionally, lncRNAs significantly contribute to the regulation of gene expression by modulating chromatin function and influencing the assembly and functioning of nuclear components, including nuclear bodies (Statello et al., 2021).

Small nuclear RNAs (snRNAs) bind to proteins in the cellular nucleus, forming complexes known as small nuclear ribonucleoproteins (snRNPs), which collaborate with other snRNAs to facilitate the maturation of mRNAs before transporting them out of the nucleus (Karijolich and Yu, 2010).

Similar to snRNAs, small nucleolar RNAs (snoRNAs) are RNA molecules residing in the nuclei of eukaryotic cells, specifically within the nucleolus, mainly encoded by intronic regions of both protein-coding and non-protein coding genes (Huang et al., 2022). These snoRNAs catalyze the 2′-O-methylation and pseudouridylation of rRNAs (Badis et al., 2003; Monaco et al., 2018), both critical post-transcriptional modifications that influence the stability, structure, and consequently the proper functioning of ribosomes (Kiss, 2002). Although mainly found in eukaryotes, there are reports of similar molecules in Archaea (Omer et al., 2000).

A subtype of snoRNAs, small Cajal RNAs (scaRNAs), are localized within Cajal bodies, nuclear organelles responsible for the biogenesis of small nuclear ribonucleoproteins (snRNPs) (Huang et al., 2022). It has been observed that scaRNAs are responsible for the 2′-O-methylation and pseudouridylation of RNA pol II-specific U1, U2, U4, and U5 spliceosomal snRNAs (Darzacq et al., 2002).

The idea that snoRNAs might be ancient remnants from the RNA world aligns with the hypothesis that early protein-coding gene exons evolved around snoRNA sequences after the emergence of templated protein synthesis (Hoeppner and Poole, 2012). However, while there is a strong argument for an RNA-world origin of snoRNAs, it is equally plausible that these RNAs have a more recent origin, evolving with the complex regulatory needs of eukaryotic cells. Further research into snoRNAs may shed light on their role in the molecular evolution of life, considering both ancient and contemporary origins (Hoeppner and Poole, 2012).

MicroRNAs (miRNAs), which are 21-23 nucleotides in size, are associated with silencing and managing gene expression through post-transcriptional regulation. miRNAs interact with a seed region, a 2 to 7 nucleotide sequence, in the 3’ untranslated region (UTR) of mRNAs, leading to translational repression through mRNA degradation (Lu and Rothenberg, 2018).

Another category of well-known RNAs is small interfering RNAs (siRNAs). These RNA molecules typically range from 21 to 23 base pairs in length. Existing as duplexes, siRNAs can downregulate gene expression. This suppression is achieved through the binding of the entire siRNA duplex to specific mRNA sequences by complementarity (Lam et al., 2015). Both miRNAs and siRNAs are small RNA-mediated regulation mechanisms (Lam e*t al.*, 2015). In an RNA world, similar molecules would have enabled primitive RNA to adapt to environmental changes and rapidly regulate expression mechanisms in response to external stimuli. This adaptation would have laid the evolutionary foundations for the development of increasingly complex gene regulation systems.

Circular RNAs (circRNAs) are covalently closed-loop RNA molecules formed through back-splicing, where an upstream 3’ splice site is joined to a downstream 5’ splice site, resulting in a circular structure (Awasthi et al., 2018). It has been shown that circRNAs act as miRNA and protein sponges, play a role in gene regulation, serve as transcriptional regulators, and act as scaffolds for proteins (Huang, et al., 2020). These circRNAs vary in length from 100 base pairs to 4 kb, depending partly on the length and number of exons they contain (Verduci et al., 2021). Their circular structure makes circRNAs less susceptible to degradation compared to linear RNAs, which have free ends. This ensures complete replication without the need for specific initiation or termination signals. Such stability and efficiency would have been advantageous in an RNA world, where circRNAs are proposed to have played a crucial role in the transition from RNA to DNA (Diener, 1989; Soslau, 2018).

Enhancer RNAs (eRNAs) are transcribed from enhancer regions of the genome. These eRNAs promote the recruitment of transcription factors and RNA polymerase II to target genes. Some studies indicate that eRNAs participate in cellular processes such as cell differentiation and development (Arnold et al., 2020). Generally, this type of non-coding RNA is less than 150 nucleotides long, but some eRNAs can be as long as 4 kb (Sartorelli and Lauberth, 2020).

Another class of non-coding RNA is piwi-interacting RNAs (piRNAs), which engage with a family of proteins known as Argonaute/Piwi. These molecules play an important role in safeguarding germline cells by preventing the insertion of transposable elements. piRNAs typically range from 21 to 35 base pairs in length and exhibit 2’-O-methyl-modified 3’ ends; this modification involves the addition of a methyl group to the 2’ hydroxyl (-OH) of the ribose moiety. Such modification extends the lifetime of alternative RNA conformational states and imparts resistance to nucleases, contributing to the overall stability and functionality of piRNAs in their protective role within germline cells (Tóth et al., 2016).

Riboswitches are another type of non-coding RNA, characterized as structured domains located within the 5’ untranslated region (UTR) of mRNAs. They consist of two distinct functional domains: an aptamer domain and an expression platform (Garst et al., 2011). These particular RNAs can switch gene expression on and off using a mechanism involving the binding of small target molecules, including metabolites (Kavita and Breaker, 2023). Riboswitches also exhibit regulatory control over the transcription of non-coding RNAs and the sequestration of the ribosome binding site (Breaker, 2018; Kavita and Breaker, 2023). Riboswitches are believed to be remnants of ancient regulatory mechanisms due to their ability to regulate gene expression independently of proteins, their distribution across all domains of life, and the fact that many riboswitches interact with RNA-derived molecules, including organic cofactors and alarmones. In this manner, riboswitches likely served sensing and regulatory functions in the RNA world, similar to their roles in present-day organisms (Breaker, 2012; Pavlova et al., 2019; Salvail and Breaker, 2023).

Other non-coding RNAs include extracellular RNAs (exRNAs), which function as signaling molecules between cells. These molecules travel via exosomes or within microvesicles and can perform long-distance regulation (Wu et al., 2022). Enhancer-associated lncRNAs (elncRNAs) are involved in the regulation of gene expression by facilitating interactions between enhancers and promoters and by recruiting transcription factors to the promoters of target genes (Hou et al., 2019). Long intergenic non-coding RNAs (lincRNAs) are independently transcribed (without directly depending on the transcription of protein-coding genes or other nearby genetic elements) molecules exceeding 200 nucleotides in length, formed from transcribed regions situated between protein-coding genes (intergenic regions). These RNAs are involved in the regulation of gene expression through different mechanisms. They can directly affect nuclear architecture, sequester intracellular molecules, or promote their function, among other roles (Ransohoff et al., 2018). 

The diversity of RNA functions is still being unraveled, with new discoveries constantly emerging. As we continue to explore the different functions of RNA in prokaryotes, eukaryotes, *in vitro* and *in silico* systems, we can expect to gain a deeper understanding of the intricate mechanisms underlying cellular processes where RNA plays a crucial role.

## Challenges and alternatives to the RNA world hypothesis

Although RNA molecules exhibit catalytic, structural, and regulatory properties and are ubiquitous in cellular processes, strongly suggesting they played a major role in the early evolution of life, the RNA world hypothesis still faces significant challenges that require thorough exploration (Bernhardt, 2012; Le Vay and Mutschler, 2019).

### Prebiotic synthesis of RNA monomers

A major hurdle for the RNA world proposal is the prebiotic synthesis of RNA monomers. Although ribonucleotides have been produced abiotically using various subsystems, common intermediates, and byproducts to link them (Powner et al., 2009; Powner and Sutherland, 2011; Sutherland, 2016), several obstacles persist. For example, there are issues related to the conditions and environments necessary for producing intermediates and byproducts, the availability of certain metals or high concentrations of compounds on early Earth, and the numerous iterations required to achieve the desired molecules (Lazcano, 2018). 

### Stability of RNA and its components

The susceptibility of RNA and its components to degradation in a dynamic environment influenced by various chemical and physical factors is a proposed counterargument against their presence on primordial Earth (Bernhardt, 2012; Xu et al., 2020). It is widely recognized that ribose, which can be formed abiotically through the formose reaction (Butlerow, 1861; Zafar and Senad, 2012; Delidovich et al., 2014; Omran et al., 2020), is unstable in strong acid or basic environments (Larralde et al., 1995). Even under neutral conditions its half-life is very short, leading to high rates of decomposition and the low availability of this sugar in a primitive environment (Larralde *et al*., 1995). 

Ultraviolet (UV) irradiation is another important factor that likely played a dual role in the prebiotic era (Cnossen et al., 2007), acting as an energy source while also causing molecular damage, thus representing a significant selective pressure in the prebiotic environment (Sagan, 1973; Ranjan and Sasselov, 2016). Single nucleobases absorb more UV light than single or double strands of nucleic acids, with single strands absorbing more than double strands (Vorlíčková et al., 2005; Winkler et al., 2020). Consequently, this makes the spontaneous synthesis of RNA difficult, as nucleobases can be degraded before they form RNA strands. However, once RNA strands are formed, their double-stranded regions are less susceptible to UV damage compared to single nucleobases, suggesting a protective advantage in RNA stability.

### Alternative pre-RNA worlds

The RNA world hypothesis posits that RNA was essential for storing genetic information and catalyzing chemical reactions in early life. However, the challenges that have been discussed here have led to the exploration of alternative pre-RNA worlds, where simpler and more chemically stable molecules might have served as the initial genetic molecules, predating RNA. These hypothetical pre-RNA molecules would not only need to possess the ability to store and replicate genetic information but also exhibit greater resilience in the dynamic environments of early Earth. The pre-RNA world proposals aim to bridge the gap between prebiotic chemistry and the sophisticated RNA-based life forms that eventually arose.

One such alternative molecule is peptide nucleic acid (PNA), a polymer similar to DNA and RNA but with a peptide-like backbone. PNA is proposed as a genetic molecule that may have preceded RNA (Nelson et al., 2000). Its backbone is composed of repeating aminoethyl glycine units linked by peptide bonds, which makes it more chemically stable than RNA and DNA (Nielsen, 2010). This stability allows PNA to resist enzymatic degradation under conditions that would typically break down RNA (Nielsen, 1993). Additionally, PNA can form stable duplexes with complementary DNA and RNA strands, illustrating its potential role in early genetic systems through PNA/DNA and PNA/RNA hybridization (Egholm et al., 1992).

Similarly, threose nucleic acid (TNA) is a nucleic acid with a threose (a four-carbon sugar) backbone that could have been an early genetic polymer (Yu et al., 2012). TNA can form stable double helices and engage in Watson-Crick base pairing with both RNA and DNA, suggesting that TNA could store genetic information and transfer it to RNA and DNA (Chaput et al., 2003). In this way, TNA could have served as an intermediate between prebiotic chemistry and the RNA world. These hypotheses highlight the potential diversity of molecules that could have preceded RNA in early evolution, enhancing our understanding of prebiotic chemistry and the transition into more complex biochemical systems.

### Transition to the modern DNA-RNA-protein world

Significant challenges and questions persist regarding the transition from the RNA world to the modern DNA-RNA-protein world (Dworkin et al., 2003; Gavette et al., 2016). In this transition, interactions between DNA, RNA, and proteins led to a specialization and division of cellular activities, with DNA becoming the primary genetic storage molecule, proteins becoming the main catalysts and structural components of the cell, and RNA retaining auxiliary functions between DNA and proteins, with regulatory, structural, and catalytic properties reminiscent of an earlier era (Cojocaru and Unrau, 2017).

One hypothesis addressing these challenges is the RNA/DNA world hypothesis. This hypothesis challenges the traditional view that DNA emerged after an RNA-based system and posits that primordial life forms possessed both RNA and DNA building blocks, with heterogeneous genetic polymers comprising both RNA and DNA sequences (Bhowmik and Krishnamurthy, 2019; Xu et al., 2020). This scenario simplifies the evolutionary transition by eliminating the need for early organisms to evolve intricate biosynthetic pathways to synthesize DNA from RNA.

Through natural selection, a progressive shift towards more homogeneous polymers would have occurred, characterized by enhanced stability, genome replication fidelity, and catalytic efficiency. This evolutionary trajectory would result in the distinct functional specialization observed in contemporary biology, where RNA primarily facilitates catalysis and information transfer, while DNA functions as the stable repository of genetic information.

### Mutual evolution of nucleic acids and proteins

Coevolution models propose that life emerged from a network of interacting molecules that evolved together to form the systems required for cellular function. This approach suggests that complex biochemical systems developed through the mutual evolution of molecules such as nucleic acids and proteins (Saad, 2018; Farías-Rico and Mourra-Díaz, 2022; Tagami and Li, 2023). 

These theories highlight the importance of molecular interactions in the origin of life, suggesting that life arose from cooperative evolution rather than a linear progression of separate systems. This likely created a dynamic environment where evolving molecules influenced each other, fostering the emergence of life from a prebiotic chemical milieu.

One aspect of this theory posits that RNA and proteins coevolved in a symbiotic relationship (Saad, 2018; Farías-Rico and Mourra-Díaz, 2022; Tagami and Li, 2023). The interdependence between these molecules could have driven the evolution of complex biochemical pathways, with the catalytic abilities of RNA complemented by the structural support provided by proteins. This cooperative relationship may have paved the way for the modern DNA-RNA-protein world.

Although coevolutionary models are attractive, the RNA world hypothesis does not exclude interactions with other types of molecules and, therefore, does not rule out the possibility of coevolution. The RNA world is completely compatible with coevolutionary models.

### Non-RNA world proposals

Other proposals that do not contemplate an RNA world include the protein-first hypothesis, which posits that polypeptides were the first self-replicating molecules, with RNA and DNA evolving later as storage media for genetic information (Andras and Andras, 2005; Bernhardt, 2012). Another hypothesis is the lipid world (Segré et al., 2001), that suggests that lipid molecules were essential precursors for primitive cell membranes. In this proposal, spontaneously formed vesicles facilitated the development of primitive metabolic networks and catalyzed chemical reactions, playing a key role in the stability and organization of early life, eventually leading to more complex cellular structures (Kahana and Lancet, 2021; Subbotin and Fiksel, 2023)

Both the protein-first and lipid world hypotheses, along with coevolution models, offer compelling alternatives to the RNA world. They highlight the diverse pathways through which life might have originated and emphasize the importance of exploring various models to fully understand the complex processes that led to the emergence of life on Earth.

## Molecular evolutionary dynamics of RNA virus and viroid quasispecies in the RNA world context

The dynamic nature of RNA viruses and the RNA world intersect in fascinating ways, bringing to light intricate evolutionary processes that have shaped the biological landscape. As previously mentioned, ribocells are hypothetical primordial cellular entities in the RNA world that use RNA, rather than DNA, as their genetic information. Today, RNA viruses and viroids are the only biological entities that use RNA to store genetic material. However RNA viruses are not considered living systems (Colón-Santos et al., 2024), they are acellular submicroscopic infectious agents that replicate inside a living host cell. RNA viruses are classified in the realm Riboviria that currently includes all RNA viruses which encode an RNA-directed RNA polymerase (ICTV, 2020). They are also classified based on their genomic structure, genome replication strategies, and evolutionary characteristics (Table 1). The genetic material of RNA viruses is characterized by a compact genome composed of ribonucleic acid which is either single-stranded (ssRNA) or double-stranded (dsRNA). The ssRNA viruses are also classified based on the polarity of their genetic material into positive-sense single-stranded RNA viruses (ssRNA+), whose genomic RNA can be directly translated into proteins by the ribosome of the cellular host (e.g., coronaviruses such as SARS-CoV-2) and negative-sense single-stranded RNA viruses (ssRNA-), which have a genomic RNA that must be transcribed into a ssRNA+ before protein synthesis (e.g., orthomyxoviruses such as influenza viruses). There are also ambisense (negative and positive) RNA viruses that translate genes from both strands (e.g., some bunyavirales) (Holmes, 2009; Hulo et al., 2011). The dsRNA viruses use their double-stranded genome as a template by the RNA-dependent RNA polymerase (RdRp) to transcribe a positive-strand RNA (e.g., reoviruses such as Rotavirus) (Hulo *et al.*, 2011). Retroviruses also have an RNA genome that uses a reverse transcriptase (RT) to transcribe RNA into DNA which is then integrated into the host genome (e.g., HIV-1) (Hulo *et al.*, 2011). RdRp and RT are essential enzymes for the replication and transcription of viral genes into mRNA in order to translate viral proteins. The RNA viral genome also has some peculiar characteristics, including secondary structure at both 3’ and 5’ ends to regulate the genome replication process, and segmentation either with a single nucleic acid molecule (monopartite) or multiple genetic fragments (multipartite) (Holmes, 2009). Moreover, some ss- and dsRNA viruses, which infect plants and fungi, have multiple genomic segments contained within different virus particles called multicomponent viruses (Holmes, 2009). Viroids, which are short (250-400 nucleotides), single-stranded, infectious, circular RNA molecules that do not encode any protein, are primarily associated with plant hosts, and replicate in the nucleus or chloroplasts via the cellular RNA polymerase II (Navarro et al., 2021) through a rolling circle mechanism (Venkataraman et al., 2021). Viroids from the family *Avsunviroidae* are endowed with hammerhead ribozymes that participate in the cleavage of oligomeric strands (Di Serio et al., 2018; 2020).

**Table 1 -:** Key features of RNA viruses.

Features	Properties
Genome architecture	Small genome size (<40 kb)
	Strandedness: Single(+ or -/ambisense) or double-stranded RNA
	Segmentation: non-segmented and segmented RNA
	Partition: mono- and multi-partite RNA
	Common overlapping genes
Genome replication	Short time replication
	Use of RNA-dependent RNA polymerase and reverse transcriptase for replication and transcription of genetic material
	Use of 3’-5’ exonuclease for proofreading
Evolutionary processes	High mutation (10^-4^-10^-6^ substitutions per nucleotide site per cell infection)
	Recombination and reassortment
	Low gene duplication
	Low horizontal gene transfer

### Viral and viroid quasispecies

RNA viruses and viroids are endowed with distinctive evolutionary features, such as short generation times, high rates of genetic recombination and reassortment, and high mutation rates (Duffy, 2018). These characteristics favor their adaptation to changes in their host environment (Dolan et al., 2018). Such an intrinsically high error rate in RNA viral populations has evolutionary implications including a high prevalence of deleterious mutations and small genomes, ranging from 2,000 to 25,000 nt, although a few may reach up to 40,000 nt (Ferron et al., 2021). According to Manfred Eigen (1971), if the number of errors surpasses the maximum tolerance for mutations of the replicating system, it can result in the generation of numerous defective phenotypes and may drive the system to extinction. RNA viruses are endowed with small genomes that, statistically speaking, are less-prone to acquire a high number of mutations, allowing them to elude Eigen’s limit. It is important to highlight that being endowed with a small genome imposes limitations on the number of proteins that a biological entity may encode. Therefore, RNA viruses maximize the use of their genome space, as the majority of their genome is protein-coding (Holmes, 2009). These evolutionary characteristics allow the generation of a large number of genetically heterogeneous viroid and RNA viral genomes by a mutation-selection process called quasispecies. This concept was adopted from the study of the origin and early evolution of life to explain the heterogeneity of primitive self-replicative macromolecules with closely related sequences at different levels of organization (hypercycles) based on the mathematical model of Eigen and Schuster (1977). According to this model, the different versions of viral genotypes are situated in a part of the genetic fitness landscape that is not particularly advantageous or disadvantageous for their survival (a flat region). In this way, these viral genotypes are more likely to outcompete other variants located in higher but narrower fitness peaks guided by natural selection. Hence, this theory describes the evolutionary dynamics of small RNA replicons with high mutation rates, such as primordial self-replicative RNAs and RNA viral genomes, as potentially-adaptable molecules to their environments. This theoretical model has experimental evidence in mutants of RNA viral populations which infect bacteria, plants, and animals (Domingo et al., 2021), as well as in *in vitro* dynamic systems used for the study of the origin of life, next- generation sequencing data, lethal mutagenesis, antiviral strategies, etc (Domingo and Schuster, 2016). 

### RNA viruses and viroids: primordial replicons?

Due to their unique and (not so) simple molecular structure and evolutionary features as dynamic heterogeneous quasispecies, RNA viruses have been proposed as relics of the RNA world. Similarly, the error-prone replication, high G+C content, and unique secondary structure of viroids are interpreted as evidence to propose that they could be primordial replicons from the RNA world (Flores et al., 2014). Indeed, a modular evolution model for the emergence of protoviroids in the precellular world has been posited, suggesting subsequent increases in genome complexity through the reorganization of modules and mutation (Flores *et al*., 2022). However, these same properties do not fully support the primordial origin of viroids. The genome size and “simplicity”, the lack of protein-coding capacity, and their dependence on plant cells exhibit significant differences to the larger, more complex, protein-coding, and self-replicative ribocells. 

Therefore, viroids are best understood as specialized RNA infectious agents that may provide insights into the minimal requirements for RNA self-replication. This reinforces the idea that RNA may have played an important role in the origin of life, but viroids should not be considered relics from the RNA world. 

Similarly, RNA viruses undergo rapid mutation, reassortment, and recombination, leading to the emergence of genetic diversity, new variants, and adaptability. This ability to evolve quickly highlights the versatility of RNA viral genomes to support the idea of the virus-first hypothesis as precellular genetic elements (Nasir et al., 2012). Additionally, some authors state that RNA viruses encode hallmark proteins crucial for genome replication, genetic expression, and morphogenesis, which have no cellular homologs. Examples of hallmark viral genes believed to have emerged from the primordial pool of primitive genetic elements in a stage called the ancient virus world are the jelly-roll capsid protein, superfamily 3 helicase, rolling circle replication initiation endonuclease, DNA primase, packaging ATPase, and RNA-dependent RNA polymerase (Koonin et al., 2006; Koonin, 2016). However, analyses based on protein structure rather than sequence have demonstrated that some of these hallmark genes, including RdRp and viral capsid proteins, are homologous to cellular components (Krupovic and Koonin, 2017; Mughal et al., 2020; Jácome et al., 2022). Different arguments contradict the idea of the primordial origin of RNA viruses in the RNA world. These include their absolute reliance on the enzymatic machinery of host cells, the cellular origin of metabolic and replication genes, and their predominance infecting eukaryotic hosts (Campillo-Balderas et al., 2015). Additionally, the complexity of cellular genomes, the polyphyletic origins of viruses, and their lack of structural components such as membranes further challenge this idea.

Viral and viroid quasispecies dynamics highlight the adaptability and resilience of RNA-based entities in the face of selective pressures. These dynamics echo the evolutionary principles that may have governed early life replicators in the RNA world. The high mutation rates observed in viral quasispecies may mirror the inherent mutability in the RNA world and the importance of genetic diversity as a driving force in evolutionary processes. However, RNA viruses and viroids are RNA-based biological entities that replicate their genetic material through different pathways, neither fully rely on ribozymes, but both are entirely dependent on cellular enzymatic machinery for their replication. Therefore, current evidence indicates that RNA viruses and viroids are not remnants of the RNA world, are not directly related to ribocells, and are unlikely to have been the primordial replicators that preceded cellular life. However, they do have similar population dynamics as quasispecies that may serve as models to help us understand the evolutionary characteristics of the early stages of life (Cruz-González et al., 2021; de la Higuera and Lázaro, 2022). 

## Conclusions

This review has highlighted the pivotal role of RNA in the early stages of life, emphasizing its extraordinary diversity of functions. 

The discoveries in the field of prebiotic chemistry, the many different roles that RNA molecules play in several cellular processes, and the participation of ribonucleotides and their derivatives in regulation, catalysis, and signaling mechanisms have changed our conception of the RNA world. 

The synthesis of RNA nucleobases and precursors, leading to the formation of short RNA oligonucleotides, appears to have been a critical step in the prebiotic era. Additionally, the emergence of an RNA-dependent RNA polymerase ribozyme capable of catalyzing its own replication would mark a significant transition from simple chemical systems to an RNA world capable of Darwinian evolution. These insights contribute to a deeper understanding of the origin and early evolution of life, as well as the evolutionary processes that led to the diversity of life forms observed today. 

Applying the quasispecies model to contemporary RNA entities, such as RNA viruses and viroids, can enhance our understanding of the evolutionary dynamics of early RNA replicons. Although determining the genomic organization of primordial RNA-based entities remains challenging with existing methodological resources, the diverse genomic architectures of current RNA viruses and viroids may provide clues about the forms adopted by RNA genomes in an RNA world.

Studying RNA and its early evolutionary roles offers valuable insights for current research in molecular biology, genetics, and biochemistry. As we continue to unravel the complexities of RNA and its functions, our understanding of the origin and early evolution of life is set to deepen, presenting exciting possibilities for future scientific exploration.
